# Integrated Prevalence Assessment of *Wuchereria bancrofti* and *Onchocerca volvulus* in Three Co-Endemic Districts of Gambella Region, Ethiopia

**DOI:** 10.4269/ajtmh.22-0392

**Published:** 2023-09-11

**Authors:** Mohammed Hassen, Aderajew Mohammed, Tekola Endeshaw, Tewodros Seid, Fikresilasie Samuel, Tadese Asmare, Henok Birhanu, Firdaweke Bekele, Adane Yayeh, Fikre Seife, Mossie Tamiru, Kadu Meribo, Zerihun Tadesse, Emily Griswold, Moses Katabarwa, Frank Richards, Gregory S. Noland

**Affiliations:** ^1^ The Carter Center, Addis Ababa, Ethiopia;; ^2^ Federal Ministry of Health, Addis Ababa, Ethiopia;; ^3^ The Carter Center, Atlanta, Georgia

## Abstract

Lymphatic filariasis (LF) and onchocerciasis (OV) are among the neglected tropical diseases (NTD) targeted for elimination in Ethiopia. We used a transmission assessment survey (TAS-1) to evaluate the serological status of OV in three co-endemic districts in Gambella simultaneously. During May and June 2019, blood samples were collected from 6- to 7-year-old children who were randomly selected through standard community-based TAS methodology. Children were tested for both circulating filarial antigen (CFA) for LF via filariasis test strip and for *Onchocerca volvulus* 16 (Ov16) antibody for OV via laboratory-based ELISA. A total of 3,377 children from 150 villages in the three districts were tested; 1,823 (54.0%) were male. All three districts had CFA results below the critical threshold for stopping LF mass drug administration (MDA). In contrast, 40 children (1.2%) were positive for Ov16 antibody, well above the WHO’s OV stop MDA threshold of 0.1%. The integrated assessment indicated two programmatic decisions: stop MDA for LF and continue MDA for OV. Accordingly, albendazole MDA was discontinued in the districts but ivermectin MDA continued. This integrated assessment showed that a random sample for TAS can give important information about OV transmission status in co-endemic areas.

## INTRODUCTION

Lymphatic filariasis (LF) and onchocerciasis (OV) are neglected tropical diseases (NTDs) caused by filarial parasites. Both are vector-borne diseases that have been targeted for global elimination.^1^ Lymphatic filariasis is caused by three species of thread-like parasitic worms, called filariae. The species *Wuchereria bancrofti* is the most common worldwide and is transmitted from person to person by several genera of mosquitoes (*Culex*, *Anopheles*, and *Aedes*). The filarial parasites in their adult stage live in the lymphatic system and have an estimated active reproductive span of 4 to 6 years. Females produce millions of small immature larvae (microfilariae) that circulate in the peripheral blood.^2^ Onchocerciasis is a disease of the skin and eye caused by *Onchocerca volvulus* that is transmitted by *Simulium* fly species (black flies) that breed in fast-flowing rivers and streams. Adult *O. volvulus* worms can live up to 14 years and are found in nodules under the skin of infected persons.^3^ Females produce microfilariae that reside in the skin and can enter the eyes, potentially causing blindness.

Lymphatic filariasis and OV are major public health problems in many tropical and subtropical countries, including Ethiopia, with significant impacts on health and quality of life. The WHO has targeted LF for elimination as a public health problem and OV for elimination of transmission. A coordinated effort has been put in place by the Ethiopian Federal Ministry of Health to achieve these goals. To eliminate LF in OV co-endemic areas where diethylcarbamazine is contraindicated, the WHO recommends MDA combining ivermectin (Mectizan, donated by Merck & Co., Inc., Rahway, NJ) and albendazole (donated by GlaxoSmithKline, Brentford, United Kingdom). The current intervention strategy for OV elimination calls for annual or semiannual MDA of ivermectin alone. Because of the shorter life spans of LF adult worms, OV programs are expected to take longer than LF programs to achieve elimination goals. Effective MDA (defined as treatment coverage of ≥ 65% total population) is expected to reduce the prevalence of *W. bancrofti* microfilariae and antigen in the blood to the levels below 1% and 2%, respectively, within 5 years. Country programs are advised to monitor MDA interventions regularly after at least five rounds of MDA and after every two to three rounds of the most recent impact assessment according to the cycles laid out by the WHO.^3^^,^^4^

Elimination of both diseases requires large-scale surveys to determine eligibility for the cessation of MDA, and a period of posttreatment surveillance thereafter. Impact assessments for both programs rely on prevalence in young children, using circulating filarial antigen (CFA) for LF and *O. volvulus* 16 (Ov16) antibody for OV. Stop-MDA thresholds differ according to the program. For LF, the threshold is significantly less than 2% CFA prevalence at the 95% confidence limit (using a decision rule based on the population size) in a sample of 6- and 7-year-old children. The sample size for such a transmission assessment survey (TAS) is generally less than 2,000 per evaluation unit. In contrast, the OV threshold is much lower at significantly less than 0.1% prevalence by Ov16 ELISA at the 95% confidence limit, requiring sample sizes of at least 3,000 children aged 5 to 9 per evaluation unit.^3^ OV programs must also demonstrate transmission interruption through entomological assessments before MDA can be stopped. We report our experience with integrating an Ov16 antibody assessment component into a standard LF TAS survey in Gambella region, Ethiopia.

The study took place in the districts (called *woredas* in Ethiopia) of Dimma, Godere, and Mengeshi, located in Gambella region in southwestern Ethiopia. This area is part of the Baro river basin. Dimma is sparsely populated and largely flat, whereas Mengeshi and Godere are hilly. These districts are all part of the Baro River Basin. All three were classified as endemic for OV in 2001; additionally, spatial modeling and earlier studies in neighboring zones and in neighboring Republic of South Sudan indicated this area was at high risk for OV transmission, and LF had already been observed in the region.^5^^–^^10^ Annual ivermectin MDA for OV began in the study districts in 2004 and continued to 2012, shifting to twice per year from 2013 onward. Once per year albendazole for LF was added starting in 2009 after LF mapping studies were completed.^10^ Reports showed that coverage of more than 65% was consistently achieved across the three districts in all rounds of MDA.

Baseline LF surveys in three districts conducted in 2009 using the immunochromatographic card test (ICT) showed antigen rates above 2% in adults.^10^ After five rounds of treatment with ivermectin and albendazole, impact assessments were done in Godere and Mengeshi in 2015. Dimma was not included in the 2015 survey because of security issues. Godere showed an acceptable reduction at the sentinel site but was above the threshold in the spot check site. As none of the districts met the eligibility criteria for full TAS, two more rounds of treatment were administered, and a follow-up survey was conducted in February 2018. Seven of 324 (2.2%) people from the sentinel site in Dimma were ICT positive but were all found negative for *W. bancrofti* microfilaria in nocturnal blood examinations. All other sites were below 2% CFA. The three districts met the criteria stated by WHO to proceed to full TAS in 2018, with parasitological and serological indicators below 1% and 2%, respectively.^4^

Onchocerciasis baseline surveys were conducted in 2001 in all three districts using skin snips from adult residents, which were examined microscopically for *O. volvulus* microfilaria (unpublished data). After more than a decade of MDA, antibody testing was introduced in Gambella region in surveys geared toward mapping the transmission zones of OV. The surveys sampled both 100 adults and 100 children under 10 years of age in each of five or six purposively sampled high-risk villages, chosen for their proximity to *Simulium* breeding sites. Adult seroprevalence was more than 10 times higher than that in children under 10 years of age across the three districts (Table 1, unpublished data).

**Table 1:** Baseline and midpoint evaluations of LF and OV prevalence in the study districts

Woreda (district)	Population, 2019	MDA started for OV/LF	LF (ICT) baseline, 2009 (*n*)	LF midpoint, 2015 (*n*)	LF pre-TAS, 2018 (*n*)	OV (mf in adults) baseline, 2001 (*n*)	Ov16 impact evaluation in adults aged ≥ 20, 2018 (*n*)	Ov16 impact evaluation in children aged 5–9, 2018 (*n*)
Dimma	29,373	2004/2009	15.9% (82)	n/a	1.4% (651)	23.0% (61)	25.0% (500)	2.4% (500)
Godere	65,929	2004/2009	29.0% (98)	1.2% (512)	0.0% (640)	37.1% (35)	27.2% (500)	1.2% (500)
Mengeshi	43,579	2004/2009	3.1% (100)	3.8% (132)	0.3% (628)	38.2% (34)	24.8% (600)	1.2% (600)

ICT = immunochromatographic card test; LF = lymphatic filariasis; MDA = mass drug administration; mf = microfilariae; OV = onchocerciasis; Ov16 = *Onchocerca volvulus* 16; TAS = transmission assessment survey.

It is recommended to integrate OV and LF impact assessments in areas where co-endemicity exists.^11^^,^^12^ Here we report results and our experience integrating an Ov16 antibody assessment into a stop-MDA LF TAS-1 survey in Gambella region, Ethiopia.

## MATERIALS AND METHODS

### Study design.

We integrated an OV impact assessment into the standard WHO LF TAS protocol.^4^ Briefly, a TAS was conducted in each district, using the implementation unit (the woreda) as the evaluation unit. Community-based surveys were conducted because primary school does not begin until age 7 in Ethiopia and school enrollment is uneven. Villages were considered the primary sampling unit and a cluster sampling method was used to evaluate each woreda independently. Sample sizes were determined using the guide in the WHO TAS manual.^13^ The Survey Sample Builder (SSB) dictated the sample size, the number of clusters, and indicated that all eligible children in the sampled clusters should be tested. Target sample sizes were 1,228 children from 55 villages in Godere, 891 children from 54 villages in Mengeshi, and 780 children from 43 villages in Dimma. The critical thresholds for positive CFA tests were 14, 11, and 9, respectively.

All residents aged 6 or 7 years who were willing to participate were eligible for inclusion. The three districts were combined into one operational transmission zone for the OV assessment, as the combined sample from the three TAS was expected to exceed the minimum sample size of 3,000 required to determine whether the < 0.1% Ov16 threshold (using the upper limit of the 95% CI) had been reached.

### Sampling and diagnostic test.

Children aged 6 or 7 years whose parents had given consent and who had themselves given assent, were tested using filarial test strips (the Alere Filariasis Test Strip [FTS], formerly Alere, Waltham, MA; now Abbott, Scarborough, ME) for LF and gave blood samples on Whatman paper number 2 for OV impact assessment. Fixed volume (75-μL) micropipettes were used to transfer whole blood samples obtained via fingerstick to the test strips. Test results were interpreted after 10 minutes according to the manufacturer’s instructions. From the same puncture, ∼100 μL of blood was placed on Whatman number 2 filter paper and set aside to dry. These dried blood spots (DBS) were packed in plastic bags with desiccant, placed in coolers, and transported to the OV molecular laboratory at the Ethiopian Public Health Institute (EPHI) in Addis Ababa. Dried blood spots were stored in refrigerators until analyzed, at which time the serum from the DBS was eluted and tested using the Onchocerciasis Elimination Program for the Americas Ov16 ELISA methodology.^14^

### Field activities.

Theoretical and practical training was held for 1 day, and three teams were organized and assigned to three districts to undertake both LF TAS and OV DBS sample collection. Each team was composed of two laboratory technologists, one EPHI and one staff member from The Carter Center–Ethiopia, and one Zonal and/or woreda NTD focal person. A supervisor, together with staff from The Carter Center–Ethiopia, oversaw the sample collection procedures and DBS storage. The laboratory technologists were responsible for sample collections, and the supervisors were engaged in communicating with the local authorities and checking the sample collection and DBS storage procedures daily. A detailed discussion was held with each woreda health office head and NTD focal person, and a tentative travel route and survey site plan was drawn with these personnel before starting the actual survey. Each kebele (ward) administration and local health extension worker were contacted ahead of time to mobilize all eligible children in the sampled villages.

### Data management and analysis.

The survey data was collected using structured questionnaires. Filariasis Test Strip results and demographic data were captured on paper and double-entered by data clerks in the office using Epi Info (version, 7.2.1.0). Analyses were done using SPSS statistical software (version 16.0; IBM, Armonk, NY). GPS coordinates were captured with advanced GPS apparatus and mapped using ArcGIS (ESRI, Redlands, CA).

## RESULTS

### Participants.

Field work took approximately 25 days in May and June 2019. We sampled 60% of all villages in the 52 kebeles in the three-district area. Different names were given for two of the same villages in Mengeshi, leading to two fewer communities sampled than specified in the SSB. Teams visited 43 (70%) of the 61 total villages in Dimma, 54 (53%) of the 101 total villages in Godere, and 53 (60%) of the 88 total villages in Mengeshi. A total of 3,377 children of aged 6 to 7 years (with 100% participation rate) living in the three districts participated in the assessment. More males than females were tested (54.0% versus 46.0%, *P =* 0.00). The majority of children were 7 years old (54.4%, *P <* 0.05).

### LF-CFA prevalence.

Of 3,377 children tested by FTS, only eight (0.2%) children were found positive for CFA. The largest number of positive children was found in Dimma district (0.6% = 6/1,002), but this was still below the critical threshold of nine. Only one positive child was found in each district of Mengeshi and Dimma districts (Table 2). None of the districts surpassed the critical threshold of maximum acceptable positive children generated by the SSB.^13^

**Table 2:** Prevalence of lymphatic filariasis CFA and antibodies to Ov16 in three districts of Gambella Region, Ethiopia, May and June 2019

District	Villages sampled	Sample size	Lymphatic filariasis		Onchocerciasis
			FTS positive, % (95% CI)	CFA critical threshold	Ov16 positive, % (95%CI)
Dimma	43	1,002	6 (0.6%) (0.3–1.3%)	9	5 (0.5%) (0.2–1.2%)
Godere	54	1,286	1 (0.08%) (0.01–0.4%)	14	28 (2.2%) (1.5–3.1%)
Mengeshi	53	1,089	1 (0.09%) (0.01–0.5%)	11	7 (0.6%) (0.3–1.3%)
Total	150	3,377	8 (0.2%) (0.1–0.5%)		40 (1.2%) (0.9–1.6%)

CFA = circulating filarial antigen; FTS = filarial test strip; Ov16 = *Onchocerca volvulus* 16.

### Ov16 antibody prevalence.

Forty of 3,377 (1.2%) DBS samples from children were positive for Ov16 antibodies, well above the WHO Stop-MDA threshold of 0.1%. The highest prevalence was found in Godere (2.2%), and the lowest prevalence was found in Dimma (0.5%). The maximum number of positive children per village was seven. No children were positive for both Ov16 and CFA.

### Geographic distribution of positive children.

**Figure 1.**
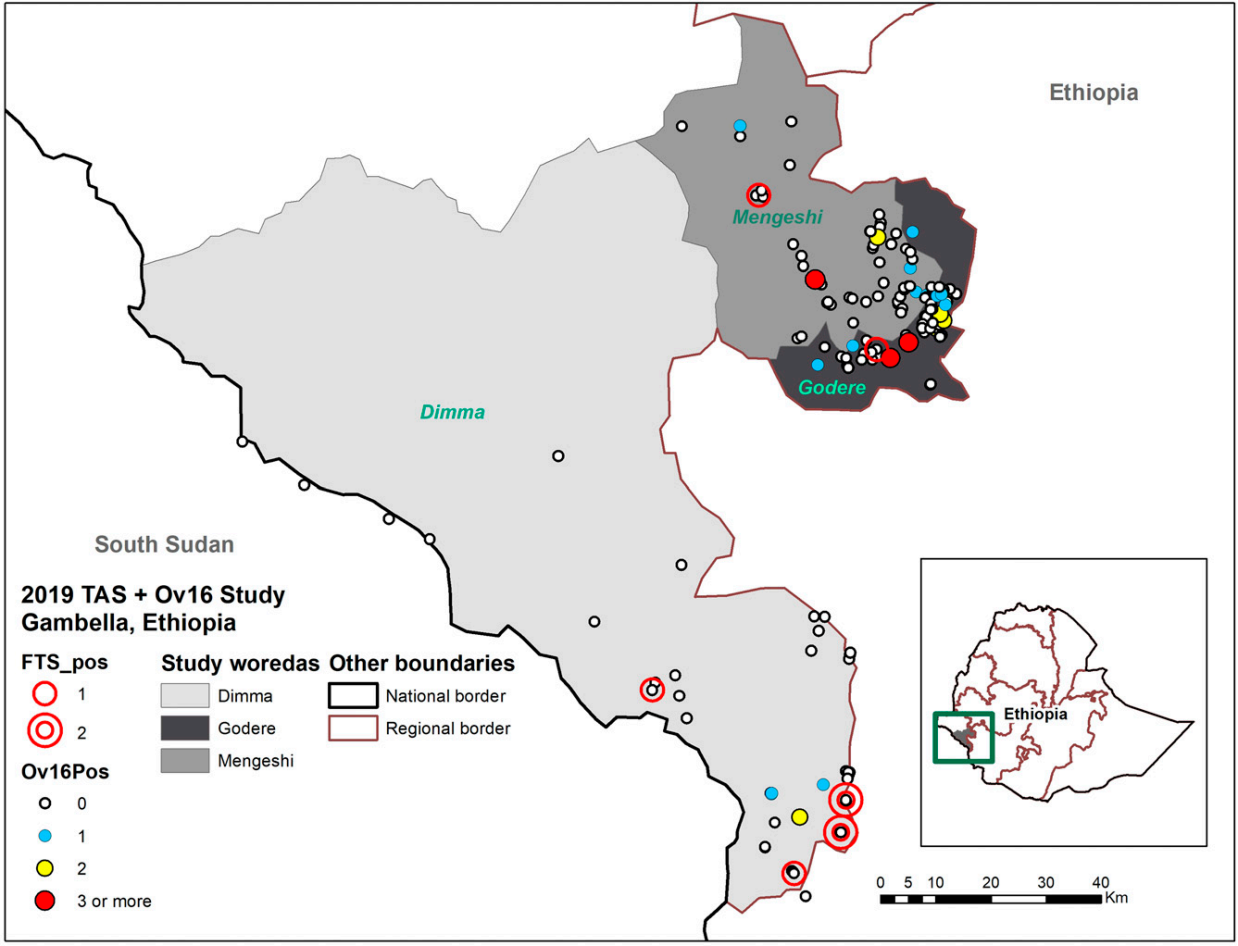
Spatial distribution of LF circulating filarial antigen and Ov16-results among children across 150 sites in three districts of Gambella Region, Ethiopia. FTS = filariasis test strip; LF = lymphatic filariasis; Ov16 = *Onchocerca volvulus* 16; pos = positive; TAS = transmission assessment survey.

Because TAS uses a random sample of communities, the selected sites should roughly align geographically with the population distribution within a given area. The maximum number of CFA-positive children per village was two. There was some clustering observed in the location of positive FTS and Ov16 tests. The majority of the FTS and Ov16-positive children in Dimma were found at the southern border, with only one FTS positive child found nearer to the center of the district (Figure 1). The districts neighboring Dimma to the south and east are also co-endemic for OV and LF, and a number of rivers flow from these higher altitude districts into Dimma. The majority of Ov16-positive children in Godere were found near the border of Mengeshi district, as were most survey sites. In Mengeshi district, five Ov16-positive children were found near the center, and the remaining three were found near the border of Godere. The Mengeshi and Godere woredas are adjacent, and thus population movements between them are common.

The Ov16-positive children in Dimma were as close as 4.5 km from a site identified as high OV risk in 2018 transmission zone mapping, based on ecological characteristics and anecdotal reports of *Simulium* breeding; however, no Ov16-positive children were found at that site at that time. Mengeshi and Godere, more densely populated, saw more even distributions of positive children. Most sites were less than 5 km from each other, and the farthest distance between positive 2019 sites and a village classified as “high risk” in 2018 was only ∼10 km. All Ov16-positive children were sampled at communities within approximately 5 km of a river, regardless of its risk classification.

## DISCUSSION

This integrated survey was the first of its kind in Ethiopia. It allowed the regional health bureau to stop MDA for LF and to recognize the need for continued MDA for OV elimination. We found that integrating an OV survey into the TAS approach made logistical sense. The addition of DBS testing added minimal time and effort to the field work and was well accepted by the communities. There were obvious efficiencies compared with conducting two separate LF and OV impact surveys. Low or absent prevalence in children born after the start of MDA should indicate that the force of infection has been brought down by the preceding years of interventions.^3^ The target age groups of TAS (6–7 years old in community-based studies) and OV Stop MDA studies (5–9 years old) partially overlap, making an integrated survey time- and resource-efficient, but the results may not be sufficiently robust in all circumstances. The TAS sample sizes are generally smaller than that required for OV serological studies to stop MDA. In our study, multiple TAS evaluation units were combined to reach the minimum OV sample size. Integrated TAS-OV surveys provided actionable data for both programs to make decisions in co-endemic areas.^12^^,^^15^^,^^16^

The study found that LF transmission had been sufficiently suppressed to stop MDA, but that OV-specific MDA needed to continue. This could be due to the longevity of *O. volvulus*, the intensity of local transmission, migration, or environmental change. Around the time of the study and afterward, efforts have been underway to improve coverage gaps, with good coverage results demonstrated elsewhere in Gambella region,^17^ and in the study districts as observed in unpublished monitoring activities.

It is imperative to note that there were four possible outcomes in this survey. First, the observed result presented in this article, where the LF prevalence by FTS was below the stop MDA critical TAS threshold, whereas the Ov16 rate was above the 0.1% stop MDA serological threshold for OV. As noted, this allowed for the important decision to stop albendazole for LF and continue ivermectin alone for OV. Second, the survey could have shown that both LF and Ov16 prevalence were above the stop MDA thresholds. In this case, the decision would be to continue with the combined albendazole and ivermectin MDA for both diseases. Third, if the survey had shown results below the “stop MDA” thresholds for both LF and OV, then the immediate decision would not be to stop all MDA. To meet all the WHO OV stop-MDA criteria might require further testing in older children up to age 9 and focused sampling in high-risk “first-line” villages near rapids of rivers; the OV evaluations would also require *Simulium* vector collections to determine whether vector infectivity rates met the WHO stop MDA criteria (O150 positivity by polymerase chain reaction < 1/2,000 flies and/or annual transmission potential ≤ 20 L3/person/year). Therefore, in effect, the treatment in this scenario would be the same as that in the first scenario: albendazole could be stopped for LF, but twice per year ivermectin for OV would need to continue, at least temporarily. Fourth, if the OV prevalence had been significantly below 0.1% but TAS failed, the program would then need to continue LF MDA annually for a minimum of two additional rounds. In the meantime, further OV stop MDA surveys could be undertaken as described earlier. If the OV program “passed” those assessments, twice per year MDA could be stopped and once per year ivermectin + albendazole treatment continued until the TAS was passed. At that point, integrated post-MDA surveillance could be undertaken.

The traditional OV stop MDA sampling focuses on communities located near fast-flowing rivers (“first-line villages”), the ecological features that favor black fly breeding and thus OV transmission. Although 60% of all villages in this area were sampled in the TAS in this exercise, theoretically the random selection of TAS could bias village selection and potentially underestimate OV prevalence, which would risk stopping OV MDA prematurely. However, in areas where habitation patterns predispose people to living near rivers, or where the distribution of vectors and their breeding sites is unknown or difficult to determine, a random sample could achieve a relatively accurate picture of OV prevalence. In this case, the random sample clearly indicated that OV transmission was ongoing. All Ov16-positive children were sampled within 5 to 6 km of a river, although none were specifically designated as high-risk communities in earlier field work; the random TAS sample did not miss these areas. The results from this study were not statistically significantly different from the 2018 Ov16 survey results for children (Table 1), where 25/1,600 (1.6%) of children from high-risk communities were positive (*P =* 0.25). However, the need for additional evaluations before stopping OV MDA should help alleviate any concerns of underestimating OV transmission in an area with different settlement patterns.

The results also hinted at a potential cross-border transmission zone for OV within Ethiopia. The positive children in Dimma woreda were concentrated in the southern part of the district near Surma, Bero, and Guraferda woredas in Southern Nations, Nationalities, and Peoples region. Movement across the regional border—either by humans or vectors—could be contributing to transmission of both OV and LF in this region.

The TAS could be tailored for results to match more readily with OV stop-MDA criteria. This could involve 1) expanding DBS/Ov16 testing to older children and/or 2) purposively sampling additional children in first-line villages in addition to the TAS sample. Depending on the context, the added costs could be marginal or considerable. Some approaches could add an unacceptable amount of time or disruption to the field work, and budgeting could be an issue if funding is directed strictly toward LF activities and traditional TAS. The large number of clusters dictated by TAS can make field work more expensive than some OV evaluations, where only a few sites are visited. However, there are savings to programs when they consider their overall fieldwork costs. Ov16 ELISA testing at a central laboratory does add significant costs and time, but these could be mitigated through the use of affordable rapid diagnostic tests (RDT), if the WHO approves OV RDTs for Stop MDA studies.^3^

Other researchers have integrated assessments for the two diseases using a variety of diagnostics. After more than 10 rounds of treatment with ivermectin only, researchers in Mali added the SD Bioline OV/LF IgG4 rapid test in two TAS evaluation units. As in our study, the criteria for passing TAS were met, but both point estimates and upper 95% confidence limits exceeded the threshold for interrupting transmission of OV.^12^ Another study in Senegal used an OV evaluation to assess LF transmission, finding ongoing transmission of both diseases.^15^ A similar study in Equatorial Guinea found minimal transmission of either disease.^18^ Elsewhere in Mali, another OV-based assessment found continuing transmission of OV but promising results for LF.^19^ The Senegal results, as well as those elsewhere in Ethiopia,^20^ emphasize the importance of using albendazole with ivermectin for the added adulticidal effect on *W. bancrofti* worms.

## CONCLUSION

Integrating an OV serological assessment into a traditional LF TAS allowed us to draw conclusions for each program within the same districts: a “stop MDA decision” for LF, but a “continue MDA” decision for OV. Although the usual and commonly practiced sampling approach for OV impact assessments is to sample children from a wider age range (5–9 years) and only those living in high-risk villages near rivers, this survey showed that a restricted age group (6–7 years) from randomly selected villages can give important information about the status of OV transmission. However, negative OV results in TAS require additional serological and entomological surveys before OV MDA can be halted. To use resources more efficiently, strengthen integrated interventions among NTD programs, and facilitate sound and prompt decision-making, it is our recommended best practice to integrate OV serological testing into all stop MDA TAS assessments in known or potentially co-endemic areas as soon as possible.
